# Constructing DNA logic circuits based on the toehold preemption mechanism†

**DOI:** 10.1039/d1ra08687a

**Published:** 2021-12-22

**Authors:** Cuicui Xing, Xuedong Zheng, Qiang Zhang

**Affiliations:** Key Laboratory of Advanced Design and Intelligent Computing, Dalian University, Ministry of Education Dalian 116622 China xingcc6535@163.com zhangq@dlut.edu.cn; College of Computer Science, Shenyang Aerospace University Shenyang 110136 China xuedongzheng@163.com; School of Computer Science and Technology, Dalian University of Technology Dalian 116024 China

## Abstract

Strand displacement technology and ribozyme digestion technology have enriched the intelligent toolbox of molecular computing and provided more methods for the construction of DNA logic circuits. In recent years, DNA logic circuits have developed rapidly, and their scalability and accuracy in molecular computing and information processing have been fully demonstrated. However, existing DNA logic circuits still have some problems such as high complexity of DNA strands (number of DNA strands) hindering the expansion of practical computing tasks. In view of the above problems, we presented a toehold preemption mechanism and applied it to construct DNA logic circuits using E6-type DNAzymes, such as half adder circuit, half subtractor circuit, and 4-bit square root logic circuit. Different from the dual-track logic expressions, all the signals in the circuits of this study were monorail which substantially reduced the number of DNA strands in the DNA logic circuits. The presented preemption mechanism provides a way to simplify the implementation of large and complex DNA integrated circuits.

## Abstract 2

Preemptor blocks the strand displacement reaction by acting on DNA complex, not by directly hybridizing with the worker.

## Graphical abstract

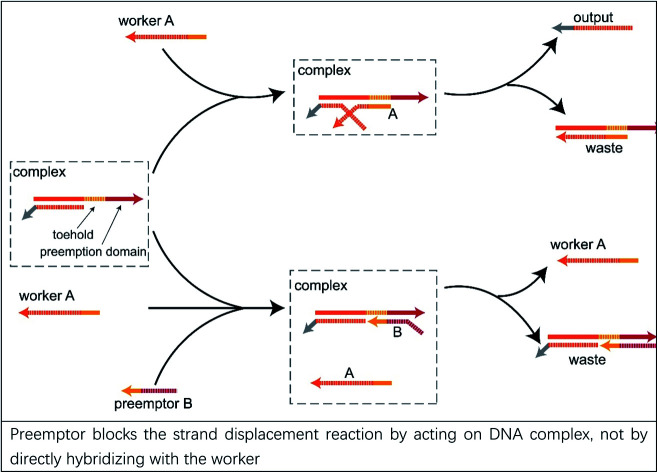

## Introduction

As a breakthrough technology of the 21st century, nano-technology has greatly promoted the development of information technology,^1–3^ biomedicine,^4–6^ environmental science,^7^ and energy science.^8^ Because of its programmability, addressability, and specificity, DNA^9,10^ has been one of the ideal materials for nanotechnology. It has been used to construct a wide variety of highly robust nanodevices, such as DNA circuits,^11–16^ arithmetic computing systems,^17^ biosensors,^18,19^ nanomachines,^20^ and probes.^21,22^ Among them, the DNA logic circuit, which can be used as the basis for the development of biological computers, has attracted extensive attention. Researchers have conducted substantial research on DNA logic circuits. As one of the most widely used technologies in the construction of DNA logic circuits, DNA strand displacement is a molecular dynamic reaction process in which the intruding strand undergoes a structural reaction with a part of the double strand substrate to replace and release the constrained single strand in the original double strand, thus generating a new double stranded structure.^23^ However, the reaction rate of DNA strand displacement between the flat-ended DNA double strand and the invading strand is very slow. In order to solve this problem, the toehold-mediated DNA strand displacement reaction has been proposed.^24–26^ The reaction rate of DNA strand displacement increases exponentially with the bonding strength, which can be increased by about 10^6^ times.^27^ Subsequently, in order to fine regulate the reaction rate of DNA strand displacement, a series of reaction regulation methods of toehold-mediated DNA strand displacement have been proposed, such as remote toehold,^27,28^ mismatch toehold,^28,29^ combination toehold,^30^ and allosteric toehold.^31^ These methods improve the flexibility of the toehold-mediated strand displacement reaction and provide a powerful tool for the development of DNA computing.^32^ In addition, in order to achieve more complex molecular logic calculation, many biological engineering methods, such as the application of restriction enzymes^33,34^ and deoxyribozymes,^35–37^ have been widely used in the construction of DNA logic circuits. Among them, deoxyribozymes or DNAzymes are the catalytic DNA sequences, which can imitate the function of proteinase. For example, the E6-type DNAzymes can rapidly catalyse continuous cleavage of the phosphate diester bond of a single RNA embedded in a complementary DNA substrate with the help of Mg^2+^ ions.^38^ Because of its high catalytic efficiency, simple synthesis and preparation, and good chemical and thermal stability, DNAzyme is particularly suitable for DNA logic operations. Substantial progress has been demonstrated, and the assembly of computational modules composed of DNAzymes has completed the operation of universal logic gates.^39^ It enables good regulation and flexible design in DNA circuits.^40,41^ The combination of the ribozyme lysis reaction and strand displacement reaction provides a new way to build DNA circuits with the ability of signal amplification and thus promotes the development of DNA logic circuits.

The scalability and accuracy of DNA logic circuits in molecular computing and information processing have been extensively demonstrated.^11–17,42–44^ Especially, nucleic acid-based constitutional dynamic networks provide versatile means to design computing circuits of enhanced complexity and advance the processing and scaling of DNA computing systems.^45^ However, existing circuits have some problems that cannot be ignored. First, most large-scale DNA circuits that have been proposed with DNA strands as inputs are dual-track circuits.^46–48^ Compared with a monorail circuit, a dual-track circuit obviously increases the complexity of DNA strands which limits the expansion of DNA logic circuit. Second, the DNA strands used as inhibitory signals do not play a very specific or unique role in the DNA logic circuit, especially the XOR gate^49–51^ in which the output suppression were realized by using complementary DNA strands as two different inputs. In this case, two complementary input DNA strands consume each other thus no output will be made. However, once the input DNA strands exerts multiple trigger functions in the DNA logic circuit, the mutual inhibition or consumption of different inputs will cause all the input strands lose their trigger functions for downstream reactions. Therefore, it is very important to establish an efficient mechanism which can be applied to DNA logic circuits to balance the trigger function and suppression function.

In this study, a toehold preemption mechanism is proposed and applied to construct DNA logic circuits using the E6-type DNAzymes. In this mechanism, the input DNA strands are independent of each other in function which means one input will not hinder another input to trigger other downstream reactions. That is, the suppression of signals does not result from the mutual suppression of the input DNA strands, but from the direct action of the input DNA strands on the DNA logic gates. To illustrate the validity of this mechanism, we created a series of basic DNA logic gates, such as an INHIBIT gate, a XOR gate, and an OR-INHIBIT gate. And by connecting basic logic gates, more complex DNA logic circuits were constructed, such as a half adder circuit, a half subtractor circuit and a 4-bit open square root circuit. These circuits are constructed with fewer DNA strands, which effectively reduces the strand complexity of DNA logic circuits and provides the possibility for the further scaling of DNA circuits.

## Materials and methods

### Materials

All DNA strands used in this study were purchased from GenScript Biotechnology Co., Ltd (Nanjing, China). Unmodified DNA strands were purified by polyacrylamide gel electrophoresis (PAGE), and DNA strands containing RNA bases or fluorescent groups were purified by high-performance liquid chromatography (HPLC). The sequences of all strands are listed in ESI Table S1.† The DNA stock solution was obtained by dissolving dry DNA powder in deionized water, and the concentration was measured at an absorbance of 260 nm using a Nanodrop 2000 spectrophotometer (Thermo Fisher Scientific Inc., USA).

### Preparation of DNA logic gates

All DNA logic gates were formed by annealing: the inhibitor strands and E6-type DNAzymes were mixed in 1x TAE/Mg^2+^ buffer (40 mM Tris, 20 mM acetic acid, 1 mM EDTA2Na, and 12.5 mM Mg(OAc)2, pH 8.0) at a molar concentration ratio of 1.2 : 1; the mixture was heated at 95 °C for 5 minutes, 65 °C for 30 min, 50 °C for 30 min, 37 °C for 30 min, 22 °C for 30 min, and preserved at 20 °C.

### Native PAGE

The sample solution (40 μL) was mixed with 60% glycerol solution in a ratio of 6 : 1 and subjected to 12% polyacrylamide gel electrophoresis in 1x TAE/Mg^2+^ buffer (40 mM Tris, 20 mM acetic acid, 1 mM EDTA2Na, and 12.5 mM Mg(OAc)2, pH 8.0) at 80 V for 160 minutes at 4 °C.

### Fluorescence signal detection

Fluorescence experiments were performed using real-time fluorescent PCR (Bio-RAD 1000) equipped with a 96-well fluorescent plate reader. The reactions were carried out in 1x TAE/Mg^2+^ buffer (40 mM Tris, 20 mM acetic acid, 1 mM EDTA2Na, and 12.5 mM Mg(OAc)2, pH 8.0) at 25 °C in a typical 40 μL reaction volume, The sample interval was 1 min.

## Results and discussion

**Fig. 1:**
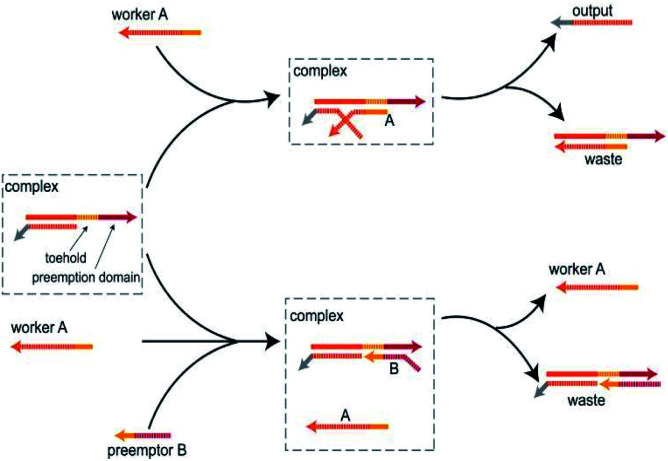
Schematic diagram of the toehold preemption mechanism. When worker A acts on complex, worker A undergoes a strand displacement reaction with the complex by toehold to produce an output. When worker A and preemptor B act on complex at the same time, preemptor B, with the help of the preemption domain, preempts toehold and makes the toehold domain unreachable, thereby preventing worker A from participating in the reaction.

As illustrated in Fig. 1, in the absence of the preemptor strand B, the worker strand A combine with the toehold and activate the branch migration resulting in the displacement of the output strand. However, although the worker strand A and the preemptor strand B share the toehold, with the help of the preemption domain, the preemptor strand B can combine with toehold much faster than the worker strand A. Thus, when the worker strand A and the preemptor strand B enter the system at the same time, the preemptor strand B preferentially bind to the DNA complex and isolate the toehold from the worker strand A, thus hindering the worker strand A to participate in the strand displacement reaction. The main feature of the toehold preemption mechanism is that the preemptor strand B blocks the strand displacement reaction triggered by the worker strand A by acting on the DNA complex, not by directly hybridizing with the worker strand A. Simply put, if the DNA complex is some computing unit, then as the two inputs, the preemptor strand B can inhibit the worker strand A without interfering with its triggering of other reaction. Moreover, the toehold preemption mechanism can simply achieve one-way inhibition and two-way cross inhibition. In this study, the toehold preemption mechanism was applied to establish DNA logic gates and assemble the DNA logic circuits using E6-type DNAzymes.

### INHIBIT gate

**Fig. 2:**
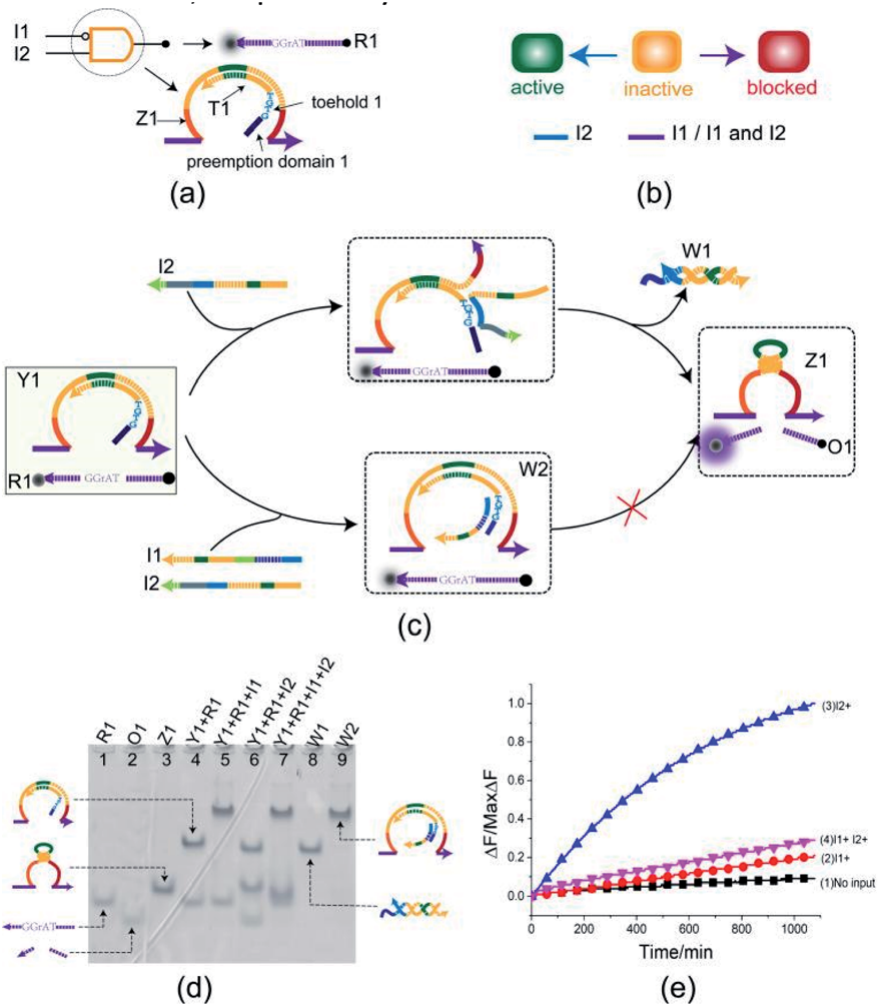
(a) Composition diagram of the INHIBIT gate. (b) The INHIBIT gate state transition diagram. The INHIBIT gate is initially in the inactive state. When input I2 acts on the INHIBIT gate, the INHIBIT gate enters the active state. When I1 (whether or not to join the input I2) acts on the INHIBIT gate, the gate enters blocked state. (c) Reaction diagram of the INHIBIT gate. The fluorophore ROX and the quencher BHQ2 are functionalized at either end of substrate strand R1. (d) Native PAGE analysis of the INHIBIT gate products. The strands and complex involved were labeled above the lane number. Lane 4, gate complex Y1 and substrate R1. Lane 5, products of INHIBIT logic gate triggered by input I1. Lane 6, products of INHIBIT logic operation triggered by input I2. Lane 7, products of INHIBIT logic gate triggered by input I1 and I2. [Y1] : [R1] : [I1] : [I2] = 1 : 1 : 1.2 : 1.2, [Y1] = 0.5 μM. (e) Time-dependent normalized fluorescence changes (Δ*F*/MaxΔ*F*) during the reaction process. Curves (1) to (4) demonstrate the gate responses to different inputs. Here, symbol + denotes the addition of strand and symbol − denotes the absence of strand. All data represent the average of three replicates. [Y1] : [R1] : [I1] : [I2] = 1 : 1 : 1.2 : 1.2, [Y1] = 0.15 μM.

The INHIBIT gate Y1 (Fig. 2(a)) is composed of two DNA strands: E6-type DNAzyme Z1 and the inhibitor strand T1 where the substrate R1 is used as a reporter strand. The gate Y1 can be activated by the input DNA strand I2 in the absence of the input DNA strand I1. Once the input DNA strand I1 is presented, the gate Y1 is blocked (Fig. 2(b)). As shown in Fig. 2(c), the DNAzyme Z1 is initially protected by the inhibitor strand T1. Once the inhibitor strand T1 is displaced from Z1 by the input strand I2, the DNAzyme Z1 is activated and cleave the free substrate R1 into two segments which can be viewed as the output. However, in the presence of the input strand I1, the strand I1 preemptively hybridize with the inhibitor strand T1 at the site of toehold 1 and preemption domain 1 which inhibit the trigger of the strand I2, thus blocking the gate and preventing the signal output. To monitor the output of the INHIBIT gate in real time, fluorescence modifications are also used by modifying fluorophore and quencher at both 5′ and 3′ ends of strand R1, respectively.

The INHIBIT gate was confirmed by PAGE gel. As shown in Fig. 2(d), the INHIBIT-gate complex Y1 coexisted with the substrate R1 in solution: two gel bands corresponding to complex Y1 and substrate R1 could be clearly observed as shown in lane 4. Upon addition of the input strand I2 to the solution, the complex Y1 and substrate R1 disintegrated which produced new gel bands corresponding to DNAzyme Z1, waste W1, and product O1 as shown in lane 6. When the input strand I1 or both I1 and I2 were added to the solution, the corresponding gel band of complex Y1 disappeared and a new corresponding gel band of complex W2 was generated as shown in lane 5 and lane 7. In addition, the gel band corresponding to substrate R1 still existed without the formation of the gel band corresponding to DNAzyme Z1 and output O1. This demonstrates the accuracy of the INHIBIT gate. It is worth noting that when the input strands I1 and I2 were both added to the solution in the same proportion, I1 first combined with gate Y1 to produce waste W2, while I2 kept free in the solution and did not participate in the reaction. The gel band corresponding to the input strand I2 is clearly visible as shown in lane 7, which proved the correctness of the toehold preemption mechanism. A fluorescence assay was also conducted to monitor the INHIBIT gate in real time. It can be clearly seen from Fig. 2(e) that fluorescence increased significantly after input strand I2 was added, while fluorescence remained at a low level when input strand I1 was added or the input strands I1 and I2 were added simultaneously.

### XOR gate

**Fig. 3:**
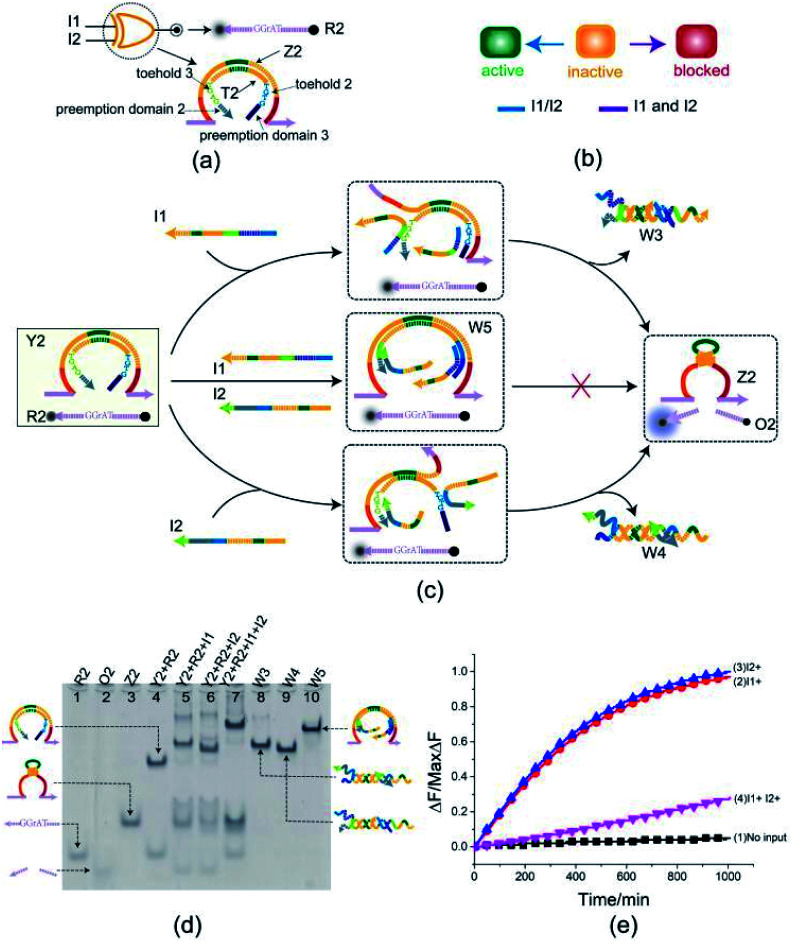
(a) Composition diagram of the XOR gate. (b) The XOR gate state transition diagram. The XOR gate is initially in the inactive state. When input I1 and I2 act on XOR gate alone, XOR gate enters the active state. When I1 and I2 act on XOR gate at the same time, XOR gate enters blocked state. (c) Reaction diagram of the XOR gate. The fluorophore FAM and the quencher BHQ1 are functionalized at either end of substrate strand R2. (d) Native PAGE analysis of the XOR gate products. The strands and complex involved were labeled above the lane number. Lane 4, gate complex Y2 and substrate R2. Lane 5, products of XOR logic gate triggered by input I1. Lane 6, products of XOR logic gate triggered by input I2. Lane 7, products of XOR logic gate triggered by input I1 and I2. [Y2] : [R2] : [I1] : [I2] = 1 : 1 : 2.4 : 2.4, [Y2] = 0.5 μM. (e) Time-dependent normalized fluorescence changes (Δ*F*/MaxΔ*F*) during the reaction process. Curves (1) to (4) demonstrate the gate responses to different inputs. Here, symbol + denotes the addition of strand and symbol − denotes the absence of strand. All data represent the average of three replicates. [Y2] : [R2] : [I1] : [I2] = 1 : 1 : 2.4 : 2.4, [Y2] = 0.15 μM.

To better test the practicality of the toehold preemption mechanism, an XOR gate was constructed. As shown in Fig. 3(a), the XOR gate Y2 is composed of two DNA strands: E6-type DNAzyme Z2 and the inhibitor strand T2 where the substrate R2 is used as a reporter strand. The gate Y2 can respond to the input strand I1 or I2, but cannot make output when the input strands I1 and I2 coexist or not (Fig. 3(b)). From Fig. 3(c), when the input strand I1 or I2 is added separately, the input strand I1 or I2 displace the inhibitor strand T2 with the help of toehold 2 or toehold 3, and then the DNAzyme Z2 is activated and cleave the free substrate R2 into two segments which can be viewed as the output. However, when the input strands I1 and I2 are added at the same time, due to the preemption domain 2 and domain 3, they inhibit each other, and the XOR gate Y2 enters a blocked state thus no signal responding.

Here, the XOR gate was validated by PAGE gel firstly as shown in Fig. 3(d). In the initial state, the gate complex Y2 and the substrate R2 can coexist in the solution which can be observed clearly as a single gel band in lane 4. When the input strand I1 (or I2) was added separately into the solution, a unit volume of input strand I1 (or I2) combined with the preemptive domain to block the gate relative to the other input strand I2 (or I1), and another unit volume of input strand I1 (or I2) triggered the gate to make a proper response by toehold-mediated DNA strand displacement. In this case, the gate complex Y2 and substrate R2 were digested (gel bands corresponding to Y2 and R2 in lane 5 or 6 disappeared) and the XOR gate made a proper response to the input (new gel bands corresponding to DNAzyme Z2, three-stranded waste W3 or W4, and product O2 can be observed clearly in lane 5 or 6). When the input strands I1 and I2 were simultaneously added to the solution in the same proportion, a unit volume of the input strands I1 and I2 were preferentially combined with the corresponding preemptive domains to block the XOR gate, so that the other unit volume of the input strands I1 and I2 did not trigger the gate. From Fig. 3(d), the top gel band in lane 7 showed the formation of four-stranded waste W5 and the gate did not make a response to the inputs I1 and I2 (no gel band corresponding to the product O2 in lane 7). The band pattern in lane 7 indicates that the preemption domain 2 (or domain 3) can preferentially bind to the input strand I1 (or the input strand I2) and make a proper competition to the toehold of the input strand I2 (or the input strand I1) thus causing the gate was blocked with respect to the input strand I2 (or the input strand I1). For the input strands I1 and I2, their mutual toehold preemption makes the gate Y2 cannot response to each of them.

To further test the validity of the toehold preemption mechanism in the XOR gate, a fluorescence assay was conducted as in shown in Fig. 3(e). For the combination of inputs (0,0), (0,1) and (1,0), the curves (blue, red, black) in Fig. 3(e) show that the gate Y2 can make proper response. For the combination of inputs (1,1), the pink curve in Fig. 3(e) illustrate the gate Y2 made no response within an acceptable range, thus indicating the toehold preemption mechanism can work properly in the XOR gate.

### OR-INHIBIT gate

**Fig. 4:**
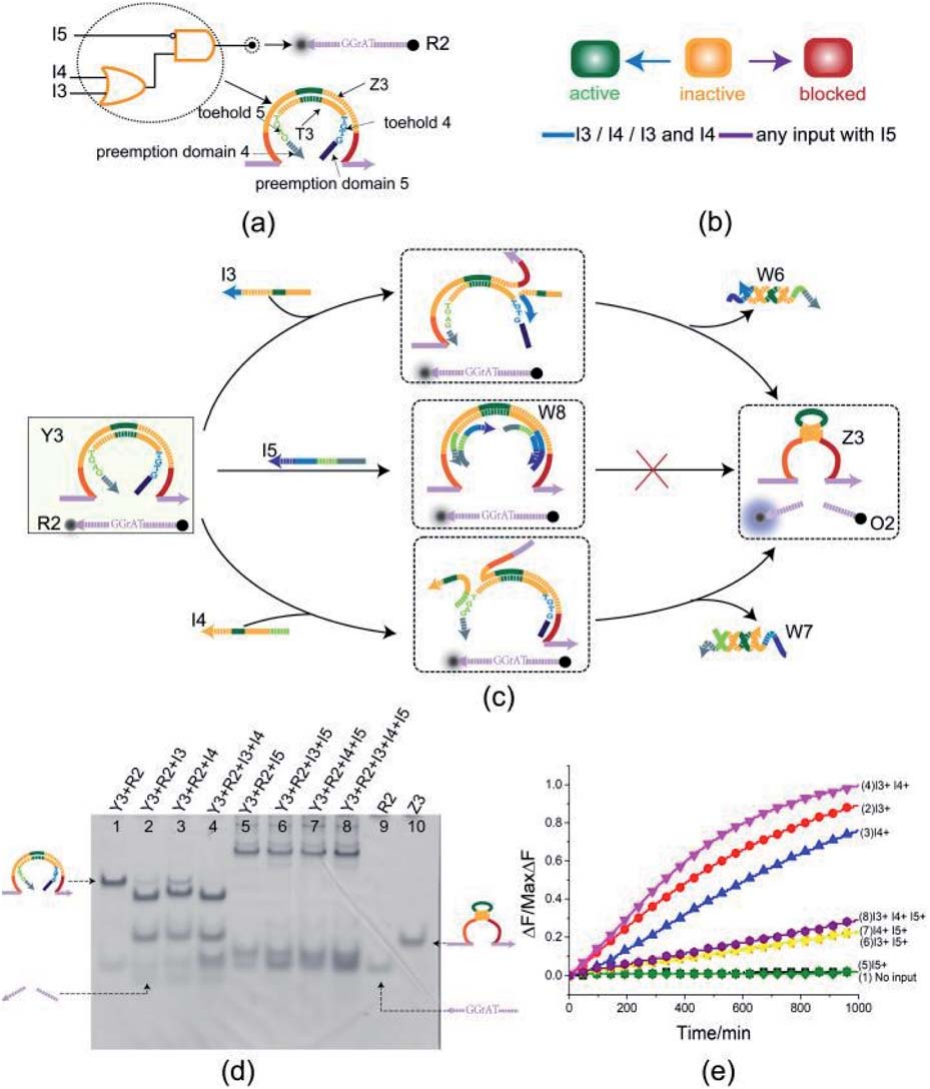
(a) Composition diagram of the OR-INHIBIT gate. (b) The OR-INHIBIT gate state transition diagram. The OR-INHIBIT gate is initially in the inactive state. When input I3 or I4 acts on OR-INHIBIT gate, OR-INHIBIT gate enters the active state. When I5 (whether or not to join the input I3 or I4) acts on OR-INHIBIT gate, OR-INHIBIT gate enters blocked state. (c) Reaction diagram of the OR-INHIBIT gate. The fluorophore FAM and the quencher BHQ1 are functionalized at either end of substrate strand R2. (d) Native PAGE analysis of the OR-INHIBIT gate products. The strands and complex involved were labeled above the lane number. Lane 1, gate complex Y3 and substrate R2. Lane 2, products of OR-INHIBIT logic gate triggered by input I3. Lane 3, products of OR-INHIBIT logic gate triggered by input I4. Lane 4, products of OR-INHIBIT logic gate triggered by input I3 and I4. Lane 5, products of OR-INHIBIT logic gate triggered by input I5. Lane 6, products of OR-INHIBIT logic gate triggered by input I3 and I5. Lane 7, products of OR-INHIBIT logic gate triggered by input I4 and I5. Lane 8, products of OR-INHIBIT logic gate triggered by input I3, I4 and I5. [Y3] : [R2] : [I3] : [I4] : [I5] = 1 : 1 : 1.2 : 1.2 : 2.4, [Y3] = 0.5 μM. (e) Time-dependent normalized fluorescence changes (Δ*F*/MaxΔ*F*) during the reaction process. Curves (1) to (8) demonstrate the gate responses to different inputs. Here, symbol + denotes the addition of strand and symbol − denotes the absence of strand. All data represent the average of three replicates. [Y3] : [R2] : [I3] : [I4] : [I5] = 1 : 1 : 1.2 : 1.2 : 2.4, [Y3] = 0.15 μM.

To illustrate the flexibility of the toehold preemption mechanism, another useful logic gate, an OR-INHIBIT gate, was established. The OR-INHIBIT gate Y3 is composed of two DNA strands (Fig. 4(a)): E6-type DNAzyme Z3 and inhibitor strand T3 where the substrate R2 is used as a reporter strand. By combining the OR gate with the INHIBIT gate, the OR-INHIBIT gate has three input strands: I3, I4, and I5. The input strands I3 and I4 are used as the OR gate inputs and the input strand I5 accompanied with the OR gate output is used as the INHIBIT gate input. The state transition diagram of the OR-INHIBIT gate is as illustrated in Fig. 4(b). The composition and structure of OR-INHIBIT gate are similar to XOR gate except different inputs. As shown in Fig. 4(c), the OR-INHIBIT gate is activated only in the presence of input strand I3 or I4. However, when the input strand I5 exists, the toehold 4 and 5 are preoccupied by the toeholds of I5 with the help of preemption domain 5 and 4 which hinders the strand displacement triggered by input strands I3 and I4, and then the OR-INHIBIT gate Y3 is blocked.

From the experimental results verified by PAGE gel (Fig. 4(d)), the OR-INHIBIT gate Y3 made proper response to the input strand I3 and I4 (lanes 2, 3 and 4). But the band pattern in lanes 5–8 shows that the input strand I5 hindered the OR-INHIBIT responding to the inputs I3 and I4 and the blocked gate did not make output. From lane 5 in Fig. 4(d), two units volume of input strand I5 combined with the gate complex Y3 to produce the waste W8 (top gel band in lane 5) and blocked the gate where the two gel bands located below in lane 5 corresponding to the substrate R2 and the excess input strand I5 was clearly visible. From lanes 6 and 7 in Fig. 4(d), under the condition that input strand I5 existed, since the gate was preferentially blocked by two units volume of input strand I5, neither input strand I3 nor input strand I4 could trigger the gate. Even when the input strand I3 and I4 existed at the same time, the gate was still blocked by the input strand I5 (lane 8 in Fig. 4(d)). The results in the fluorescence assay (Fig. 4(e)) showed that the OR-INHIBIT worked as expected: the fluorescence values increased significantly only when the input strand I3 or I4 was added, and the fluorescence values remained at low levels for all other inputs.

### Half adder

**Fig. 5:**
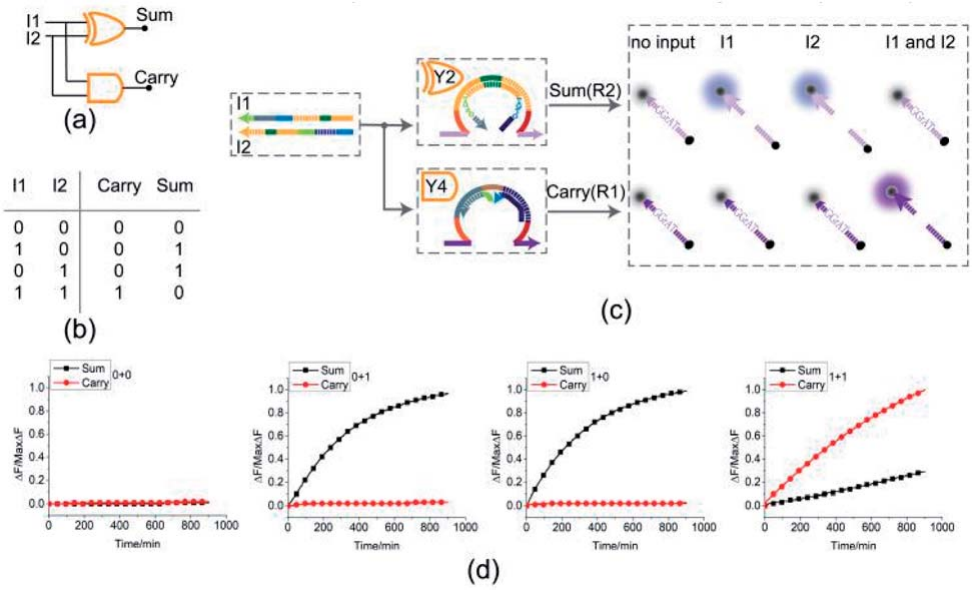
(a) Logic gate diagram of the half adder. (b) Truth table. (c) Reaction diagram of the half adder. The fluorophore FAM and the quencher BHQ1 are functionalized at either end of substrate strand R2. The fluorophore ROX and the quencher BHQ2 are functionalized at either end of substrate strand R1. (d) Time-dependent normalized fluorescence changes (Δ*F*/MaxΔ*F*) during the calculation process. All data represent the average of three replicates. [Y2] : [Y4] : [R1] : [R2] : [I1] : [I2] = 1 : 1 : 1 : 1 : 3.6 : 3.6, [Y2] = 0.15 μM.

To test the possibility of assembling DNA logic circuits, a half adder was established by combining an AND gate and a XOR gate (Fig. 5(a)). Corresponding to the truth table in Fig. 5(b), the half adder was constructed by juxtaposing the AND gate Y4 (Fig. S1†) and the XOR gate Y2 as shown in Fig. 5(c). For the input strands I1 and I2, the XOR gate Y2 is responsible for the sum portion of the half adder and the AND gate Y4 is responsible for the carry portion of the half adder. For the independence of each other in triggering the XOR gate, the input strands I1 and I2 can still activate the AND gate Y4 when they coexist in the solution. The half adder can work by mixing the XOR gate Y2 and the AND gate Y4. Our complete half adder circuit consisted of only two substrates, making it very compact.

The results of fluorescence assay are shown in Fig. 5(d). From left to right, each diagram illustrates an experimental result with a specific combination of inputs. The output of the XOR gate (Sum) and the AND gate (Carry) were expressed by the fluorescence variations of FAM (black curve) and ROX (red curve), respectively. From Fig. 5(d), corresponding to the calculation 0 + 0 = 00, the fluorescence intensity of FAM and ROX was unchanged when no inputs were added into the solution (the first diagram from the left). When the input strand I1 was added into the solution, the half adder would perform the proper calculation 1 + 0 = 01 as shown in the second diagram from the left. The similar results of the calculation 0 + 1 = 01 can be seen in the third diagram. When input strands I1 and I2 were added into the solution at the same time, the fluorescence intensity of FAM remained at a low level, but that of ROX increased significantly, resulting in the calculation of 1 + 1 = 10 (the fourth diagram from the left). The results of PAGE gel experiment (as in shown in ESI Fig. S4†) are completely consistent with the results of the fluorescence experiment, further testing the validity of the half adder circuit.

### Half subtractor

**Fig. 6:**
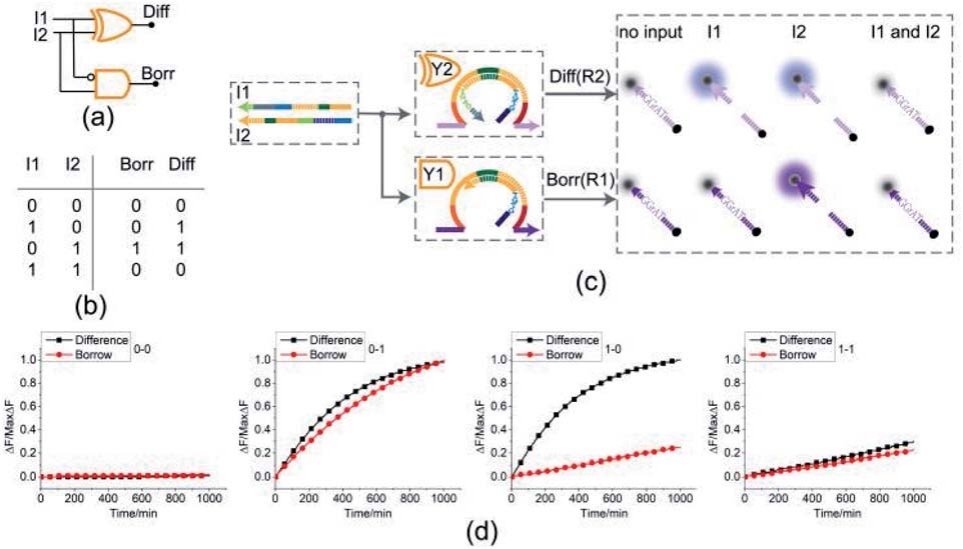
(a) Logic gate diagram of the half subtractor. (b) Truth table. (c) Reaction diagram of the half subtractor. The fluorophore FAM and the quencher BHQ1 are functionalized at either end of substrate strand R2. The fluorophore ROX and the quencher BHQ2 are functionalized at either end of substrate strand R1. (d) Time-dependent normalized fluorescence changes (Δ*F*/MaxΔ*F*) during the calculation process. All data represent the average of three replicates. [Y2] : [Y1] : [R1] : [R2] : [I1] : [I2] = 1 : 1 : 1 : 1 : 3.6 : 3.6, [Y2] = 0.15 μM.

By combining a XOR gate with an INHIBIT gate, a standard half subtractor model can be established as shown in Fig. 6(a). The truth table for the standard half subtractor model is shown in Fig. 6(b). Similar to the half adder, the half subtractor model is realized by juxtaposing the XOR gate Y2 and the INHIBIT gate Y1 (Fig. 6(c)) where the XOR gate Y2 is responsible for the difference portion of the half subtractor and the INHIBIT gate Y1 is responsible for the borrow portion of the half subtractor. We mixed the constructed XOR gate Y2 and the INHIBIT gate Y1 in the same solution, resulting in a half subtractor. Our complete half subtractor is as compact as the half adder and consisted of only two substrates.

The fluorescence assay was conducted as shown in Fig. 6(d) where the outputs of the XOR gate (Diff) and the INHIBIT gate (Borr) were expressed by fluorescence variations of FAM and ROX, respectively. In Fig. 6(d), each diagram illustrates an experimental result with a specified combination of inputs. The fluorescence intensity for FAM and ROX remained at low state when no inputs were present, which meant the calculation result was 0–0 = 00 (the first diagram from left). When only the input strand I1 was added into the solution, the output of XOR gate was true, and the output of INHIBIT gate was false. The calculation result of 1–0 = 01 was thus displayed as shown in the second diagram from left. When calculating 0–1, the input strand I2 was added into solution individually, the fluorescence intensities of FAM and ROX both increased significantly. A calculation result of 0–1 = 11 was displayed in the third diagram from left. When the two input strands I1 and I2 coexisted in the solution, both the XOR gate and INHIBIT gate made no response to the inputs within an acceptable range, meaning a calculation result of 1–1 = 00 (the fourth diagram from left). The result of PAGE gel experiment result can be seen in ESI Fig. S5.†

### 4-bit open square root circuit

**Fig. 7:**
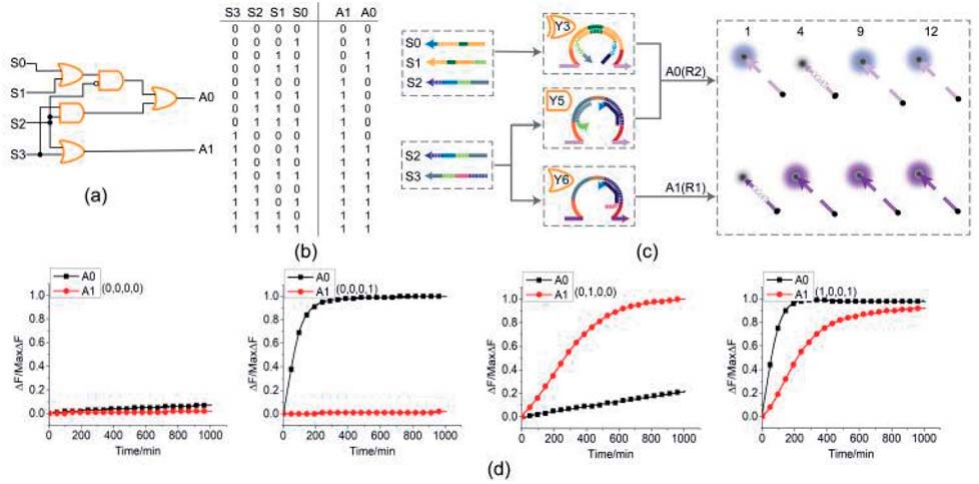
(a) Logic gate diagram of the 4-bit open square root circuit. (b) Truth table. (c) Reaction diagram of the open square root circuit. The fluorophore FAM and the quencher BHQ1 are functionalized at either end of substrate strand R2. The fluorophore ROX and the quencher BHQ2 are functionalized at either end of substrate strand R1. (d) Time-dependent normalized fluorescence changes (Δ*F*/MaxΔ*F*) during the calculation process. All data represent the average of three replicates. [Y3] : [Y5] : [Y6] : [R1] : [R2] : [S0] : [S1] : [S2] : [S3] = 1 : 1 : 1 : 1 : 1 : 1 : 1 : 3.6 : 2.4, [Y3] = 0.15 μM.

To demonstrate the ability of assembling large-scale logic circuits, a 4-bit open square root DNA circuit to compute the floor of the square root was established. The diagram of logic circuit is shown in Fig. 7(a) and the corresponding truth table is shown in Fig. 7(b). As shown in Fig. 7(c), the 4-bit open square root circuit was constructed by juxtaposing three DNA logic gates: an OR-INHIBIT gate Y3, an AND gate Y5 and an OR gate Y6. Corresponding to the truth table, the outputs of OR-INHIBIT gate Y3 and AND gate Y5 are responsible for the A0 portion of the open square root circuit, while the output of OR gate Y6 is responsible for the A1 portion. The outputs of the OR-INHIBIT logic gate and the AND logic gate (A0) were expressed by fluorescence variations of FAM while the output of the OR gate (A1) was expressed by fluorescence variations of ROX. The 4-bit open square root circuit can work by mixing the three DNA logic gates in the same solution and only three substrates are used thus making the circuit very compact.

The computing kinetics of four representative inputs that lead to different outputs are shown in Fig. 7(d) (more fluorescence experimental results can be seen in ESI Fig. S6(b)†). In the first diagram from left in Fig. 7(d), the fluorescence intensities of FAM and ROX remained at low level when no inputs were present, which meant the calculation result was 
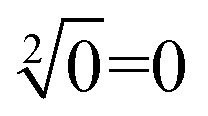
. When only input strand S0 was added into the solution, the OR-INHIBIT gate outputted true, and the AND gate and the OR gate outputted false. The calculation result of 
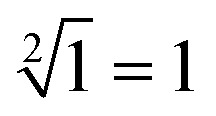
 was thus displayed in the second diagram from left in Fig. 7(d). When calculating the square root of 4, the input strand S2 was added into solution individually, the fluorescence intensities of ROX increased significantly while the fluorescence intensities of FAM remained at a low level. A calculation result of 
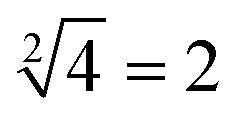
 was displayed in the third diagram from left in Fig. 7(d). When calculating the floor of the square root of 9, input S0 and S3 were added into solution at the same time, the fluorescence intensities of FAM and ROX increased significantly. The calculation result of 
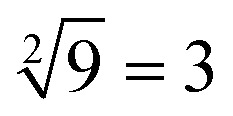
 was displayed in the fourth diagram from left in Fig. 7(d). The PAGE gel experimental results, completely consistent with the results of the fluorescence experiment, are shown in ESI Fig. S6(a).†

With the juxtaposition of three DNA logic gates, we created a 4-bit open square root DNA logic circuit to compute the floor of square root. With only three DNA logic gates, the 4-bit open square root DNA circuit illustrated a reduction of in the computing units. Compared with the similar DNA circuits^43,47^ established by the toehold-mediated DNA strand displacement, only 14 DNA strands participated the computation in our created 4-bit open square root DNA circuit which reduced the strand complexity significantly.

## Conclusions

In this study, we have proposed a toehold preemption mechanism to achieve signal inhibition and applied it to the E6-type DNAzymes. A series of basic DNA logic gates, namely INHIBIT gate, XOR gate and OR-INHIBIT gate, were constructed. In addition, three kinds of large-scale monorail circuits, namely half adder, half subtractor, and 4-bit open square root circuits, were constructed in a modular and controllable manner. These three circuits were built with less than 15 DNA strands, which greatly reduced the strand complexity of large-scale DNA logic circuits. The experimental results showed that the DNA logic circuits were well implemented and reliable. Besides, the full adder has been attempted and its reliable operation still requires further optimization in the design. Whether the basic logic circuit can be scaled up is of great significance for the construction of complex biochemical reaction circuits. However, the main difficulty in the operation of scaled-up systems is that toehold mediated activating of the double-stranded gate structures is kinetically affected by the toehold length and the DNAzyme conformation. We are still looking for methods to improve the structure of the DNAzyme-based gates. The toehold preemption mechanism can be applied to any strand displacement reaction mediated by toehold, indicating that this strategy provides a common strategy for constructing DNA circuits and can be applied to signal control or signal processing equipment. The construction of large-scale monorail circuits leads us to believe that the toehold preemption mechanism has the potential to help build more complex and concise molecular computing systems. It could open up more concepts and strategies for dynamic molecular control, which could lead to the development of emerging nanomachines, biosensors, and disease diagnostics.

To construct sophisticated biochemical circuits from scratch.

## Conflicts of interest

There are no conflicts to declare.

## Supplementary Material

RA-012-D1RA08687A-s001
