# Supplementary material for: Constructing DNA logic circuits based on the toehold preemption mechanism
Source: RSC Adv. 2021 Dec 22;12(1):338–45. doi: 10.1039/d1ra08687a (PMC8978688; doi:10.1039/d1ra08687a)
Supplement: RA-012-D1RA08687A-s001 [file RA-012-D1RA08687A-s001.pdf]

Electronic Supplementary Material (ESI) for RSC Advances.
This journal is © The Royal Society of Chemistry 2021

Supplementary Materials

Constructing DNA logic circuits based on the toehold preemption mechanism

Cuicui Xing [a‡], Xuedong Zheng [b‡] and Qiang Zhang [a,c*]

[a] Key Laboratory of Advanced Design and Intelligent Computing, Dalian University, Ministry of Education, Dalian 116622, China; xingcc6535@163.com

[b] College of Computer Science, Shenyang Aerospace University, Shenyang 110136, China; xuedongzheng@163.com

[c] School of Computer Science and Technology, Dalian University of Technology, Dalian 116024, China; zhangq@dlut.edu.cn

‡ These authors contributed equally to the article

* Correspondence: Prof. Qiang Zhang, Email: zhangq@dlut.edu.cn; Tel.: +8641187403733(Q. Z.)

Contents

Supplementary Materials 1
Constructing DNA logic circuits based on the toehold preemption mechanism 1
S1：Basic logic gate 3
S1.1 AND-H gate..3
S1.2 AND-K gate 4
S1.3 OR gate..5
S2: Large scale circuit 6
S2.1Half adder...6
S2.2 Half subtractor 8
S2.3 4-bit open square root circuit 9
S3.DNA sequences 11

S1：Basic logic gate

S1.1 AND-H gate

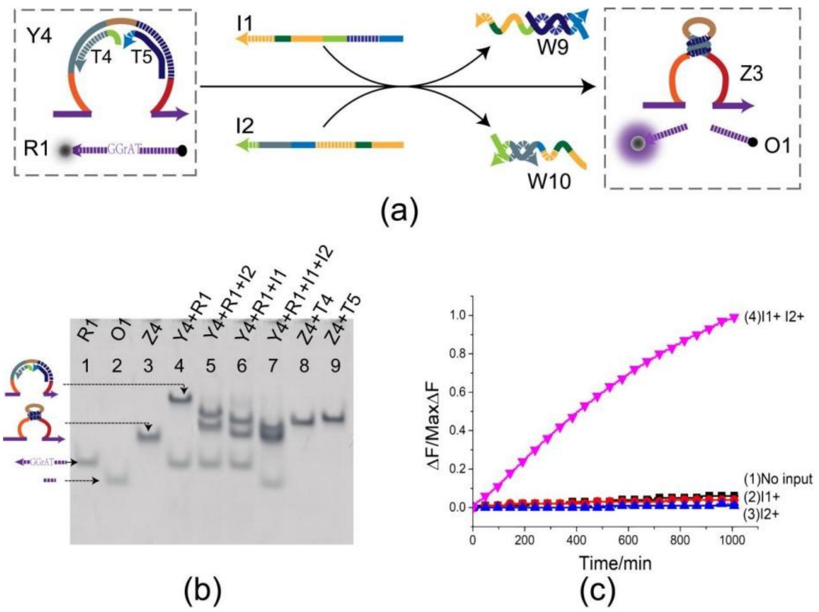

Figure S1. (a) The composition diagram and reaction diagram of the AND-H gate. The fluorophore ROX and the quencher BHQ2 are functionalized at either end of substrate strand R1. (b) Native PAGE analysis of the AND-H gate products. The strands and complex involved were labeled above the lane number. Lane 4, gate complex Y4 and substrate R1. Lane 5, products of AND-H logic gate triggered by input I2. Lane 6, products of AND-H logic operation triggered by input I1. Lane 7, products of AND-H logic gate triggered by input I1 and I2. [Y4]:[R1]:[I1]:[I2]=1:1:1.2:1.2, [Y4]=0.5μM. (c) Time-dependent normalized fluorescence changes (ΔF/MaxΔF) during the reaction process. Curves (1) to (4) demonstrate the gate responses to different inputs. Here, symbol + denotes the addition of strand and symbol - denotes the absence of strand. All data represent the average of three replicates. [Y4]:[R1]:[I1]:[I2]=1:1:1.2:1.2, [Y4]=0.15μM.

The AND-H gate Y4 (Figure S1(a)) is composed of three strands: E6-type DNAzyme Z4 and the inhibitor strand T4 and T5 where the substrate R1 is used as a reporter strand. the DNAzyme Z4 is initially protected by the inhibitor strands T4 and T5. Once the inhibitor strand T4 and T5 are displaced from Z4 by the input strand I1 and I2, the DNAzyme Z4 is activated and cleave the free substrate R1 into two segments which can be viewed as the output. However, in the presence of the input strand I2 (or I1) alone, the DNAzyme Z4 still remian inactiveted. To monitor the output of the AND-H gate in real time, fluorescence modifications are also used by

modifying fluorophore and quencher at both 5’ and 3’ ends of strand R1, respectively.

The AND-H gate was confirmed by PAGE gel. As shown in Figure S1(b), the AND-H-gate complex Y4 coexisted with the substrate R1 in solution: two gel bands corresponding to complex Y4 and substrate R1 could be clearly observed as shown in lane 4. Upon addition of the input strand I1 (or I2) to the solution, the complex Y4 disintegrated which produced new gel bands corresponding to complex Z4/T5 (or Z4/T4) and waste W9 (or W10) as shown in lane 6 (or 5). In addition, the gel band corresponding to substrate R1 still existed without the formation of the gel band corresponding to DNAzyme Z4 and output O1. When the input strand I2 and I1 were added to the solution, the complex Y4 and substrate R1 disintegrated which produced new gel bands corresponding to DNAzyme Z4, waste W9, waste W10, and product O1 as shown in lane 7. This demonstrates the accuracy of the AND-H gate. A fluorescence assay was also conducted to monitor the AND-H gate in real time. It can be clearly seen from Figure S1(c) that fluorescence increased significantly after input strand I1 and I2 were added simultaneously, while fluorescence remained at a low level when input strand I1 or I2 was added separately.

S1.2 AND-K gate

As shown in Figure S2(a), The AND-K gate Y5 is composed of three strands: DNAzyme Z5 and the inhibitor strands T6 and T7 where the substrate R2 is used as a reporter strand. The gate Y5 can response to the input strand S2 and S3 together, but can not make output when the input strands S2 or S3 present alone.

The AND-K gate was confirmed by PAGE gel. As shown in Figure S2(b), the AND-K-gate complex Y5 coexisted with the substrate R2 in solution: two gel bands corresponding to complex Y5 and substrate R2 could be clearly observed as shown in lane 4. Upon addition of the input strand S2 (or S3) to the solution, the complex Y5 disintegrated which produced new gel bands corresponding to complex Z5/T7 (or Z5/T6) and waste W11 (or W12) as shown in lane 5 (or 6). In addition, the gel band corresponding to substrate R2 still existed without the formation of the gel band

corresponding to DNAzyme Z5 and output O2. When the input strand S2 and S3 were added to the solution, the complex Y5 and substrate R2 disintegrated which produced new gel bands corresponding to DNAzyme Z5, waste W11, waste W12, and product O2 as shown in lane 7. This demonstrates the accuracy of the AND-K gate. A fluorescence assay was also conducted to monitor the AND-K gate in real time. It can be clearly seen from Figure S2(c) that fluorescence increased significantly after input strand S2 and S3 were added simultaneously, while fluorescence remained at a low level when input strand S2 or S3 was added separately.

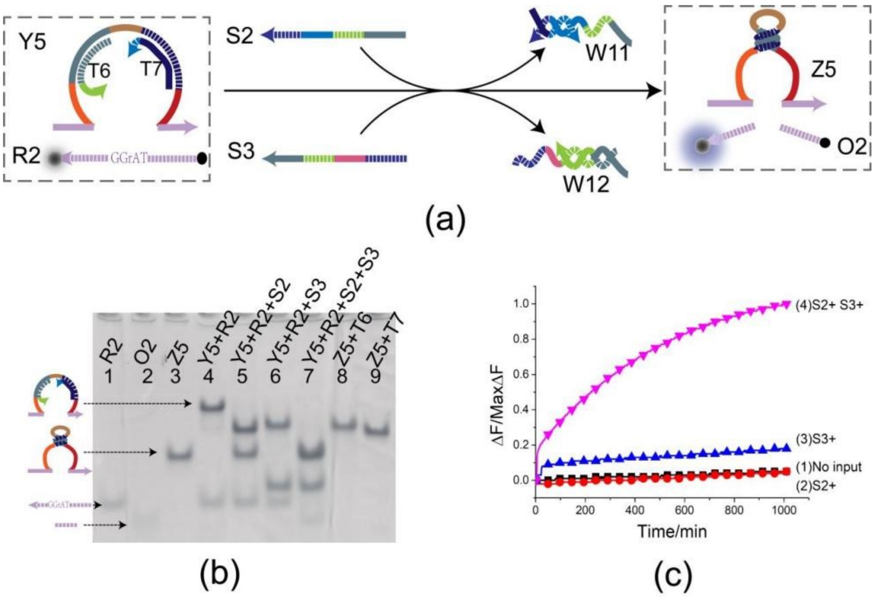

Figure S2. (a) The composition diagram and reaction diagram of the AND-K gate. The fluorophore FAM and the quencher BHQ1 are functionalized at either end of substrate strand R2. (b) Native PAGE analysis of the AND-K gate products. The strands and complex involved were labeled above the lane number. Lane 4, gate complex Y5 and substrate R2. Lane 5, products of AND-K logic gate triggered by input S2. Lane 6, products of AND-K logic operation triggered by input S3. Lane 7, products of AND-K logic gate triggered by input S2 and S3. [Y5]:[R2]:[S2]:[S3]=1:1:1.2:1.2, [Y5]=0.5μM. (c) Time-dependent normalized fluorescence changes (ΔF/MaxΔF) during the reaction process. Curves (1) to (4) demonstrate the gate responses to different inputs. Here, symbol + denotes the addition of strand and symbol - denotes the absence of strand. All data represent the average of three replicates. [Y5]:[R2]:[S2]:[S3]=1:1:1.2:1.2, [Y5]=0.15μM.

S1.3 OR gate

The OR gate Y6 is composed of two strands (Figure S3(a)): DNAzyme Z6 and the inhibitor strand T8 where the substrate R1 is used as a reporter strand. the DNAzyme

Z6 is initially protected by the inhibitor strand T8. Once the inhibitor strand T8 is displaced from Z6 by the input strand S2 or S3, the DNAzyme Z6 is activated and cleave the free substrate R1 into two segments which can be viewed as the output.

From the experimental results verified by PAGE gel (Figure S3(b)), the OR gate Y6 made proper response to the input strand S2 or S3 (lanes 5, 6 and 7). The results in the fluorescence assay (Figure S3(c)) showed that the OR gate worked as expected: the fluorescence values increased significantly when the input strand S2 or S3 was added, and the fluorescence values remained at low levels when no input was added.

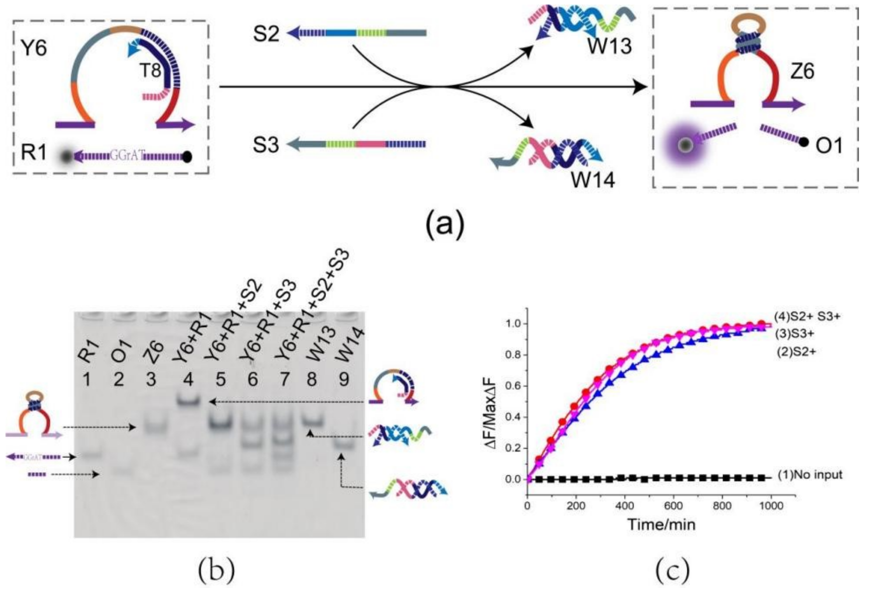

Figure S3. (a) The composition diagram and reaction diagram of the OR gate. The fluorophore ROX and the quencher BHQ2 are functionalized at either end of substrate strand R1. (b) Native PAGE analysis of the OR gate products. The strands and complex involved were labeled above the lane number. Lane 4, gate complex Y6 and substrate R1. Lane 5, products of OR logic gate triggered by input S2. Lane 6, products of OR logic operation triggered by input S3. Lane 7, products of OR logic gate triggered by input S2 and S3. [Y6]:[R1]:[S2]:[S3]=1:1:1.2:1.2, [Y6]=0.5μM. (c) Time-dependent normalized fluorescence changes (ΔF/MaxΔF) during the reaction process. Curves (1) to (4) demonstrate the gate responses to different inputs. Here, symbol + denotes the addition of strand and symbol - denotes the absence of strand. All data represent the average of three replicates. [Y6]:[R1]:[S2]:[S3]=1:1:1.2:1.2, [Y6]=0.15μM.

S2: Large scale circuit

S2.1Half adder

The half adder is composed of two gates (Figure 5(c)): a XOR gate and an AND-H

gate where the substrates R1 and R2 are used as reporter strands. The output of the XOR logic gate was the sum portion of the half adder gate. The output of the AND-H logic gate was the carry portion of the half adder gate.

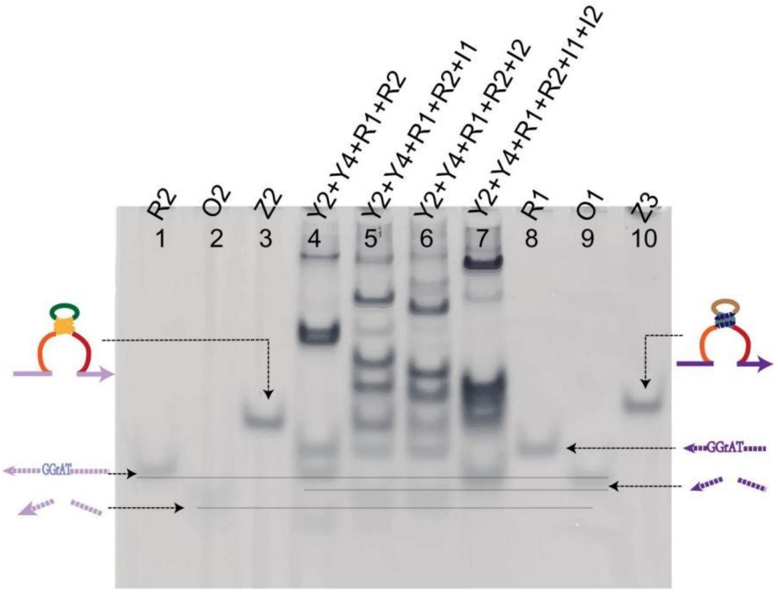

Figure S4. Native PAGE analysis of the half adder products. The strands and complex involved were labeled above the lane number. Lane 4, gate complex Y2, Y4 and substrate R1, R2. Lane 5, products of the half adder circuit triggered by input I1. Lane 6, products of the half adder circuit triggered by input I2. Lane 7, products of the half adder circuit triggered by input I1 and I2. [Y2]:[Y4]:[R1]:[R2]:[I1]:[I2]=1:1:1:1:3.6:3.6, [Y2]=0.5μM.

To verify the correctness of the experiment, the electrophoresis gel imaging experiment was conducted, and the result was shown in Figure S4. In the absence of any input, Y2, Y4, R1 and R2 coexisted in the solution. Gel bands corresponding to substrate R1 and R2 were clearly visible (as shown in lane 4), indicating 0+0=00. When I1 was added to the solution, T2 and T4 were displaced, forming double-stranded waste, DNAzyme Z2 was activated, substrate R2 was cut, and the product O2 was generated, but DNAzyme Z4 still bound to T5 and could not be activated, R1 still existed and no product O1 was produced. The gel band corresponding to R2 disappeared, and the gel band corresponding to O2 generated (as shown in lane 5), indicating 1+0=01. When input I2 was added to the solution, T2 and T5 were displaced. DNAzyme Z2 was activated and substrate R2 was cut to produce product O2. However, DNAzyme Z4 was still combined with T4 and could not be activated. R1 still existed and no product O1 was generated. The gel band corresponding to R2 disappeared, and the gel band corresponding to O2 was generated (as shown in lane 6), indicating 0+1=01. When input I1 and I2 were added to the solution at the same time, T4 and T5 were displaced, DNAzyme Z4 was activated, substrate R1 was cut and product O1 generated, but DNAzyme Z2 was still combined with T2 and could not be activated, R2 still existed and no product O2 generated. The gel band corresponding to R1 disappeared, and the gel band corresponding to O1 generated (as shown in lane 7), indicating 1+1=10. The

experimental results were in good agreement with the truth table, which verifies the accuracy of the experiment.

S2.2 Half subtractor

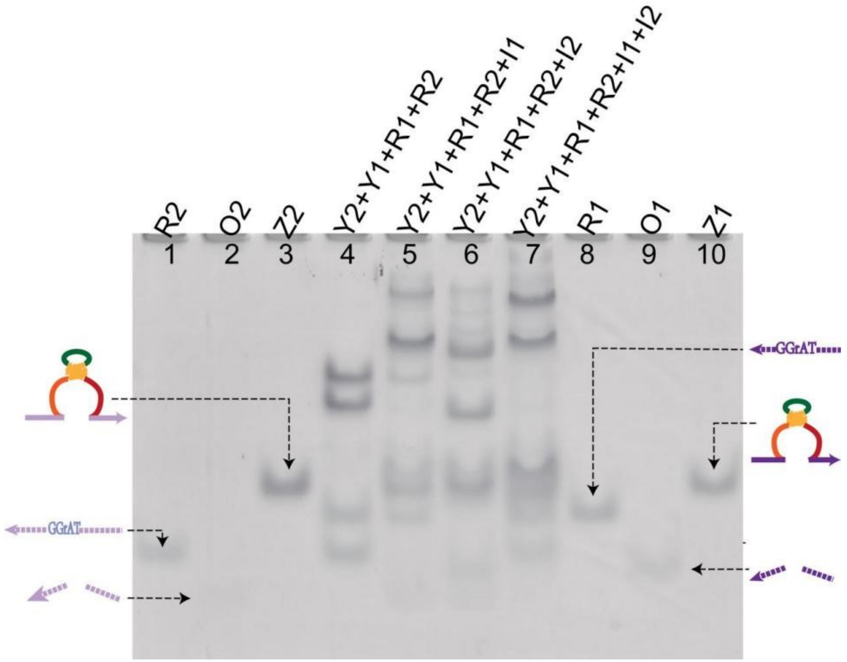

Figure S5. Native PAGE analysis of the half subtractor products. The strands and complex involved were labeled above the lane number. Lane 4, gate complex Y1, Y2 and substrate R1, R2. Lane 5, products of the half subtractor circuit triggered by input I1. Lane 6, products of the half subtractor circuit triggered by input I2. Lane 7, products of the half subtractor circuit triggered by input I1 and I2. [Y2]:[Y1]:[R1]:[R2]:[I1]:[I2]=1:1:1:1:3:3, [Y1]=0.5μM.

The half subtractor is composed of two gates (Figure 6(c)): a XOR gate and an INHIBIT gate where the substrates R1 and R2 are used as reporter strands. The output of the XOR logic gate was the difference portion of the half subtractor gate. The output of the INHIBIT logic gate was the borrow portion of the half subtractor gate.

To verify the accuracy of the experiment, an electrophoretic gel imaging experiment was carried out, and the experimental results were shown in Figure S5. In the absence of any input, Y1, Y2, R1 and R2 coexisted in the solution, and substrate R1 and R2 could not be cut. Gel bands corresponding to R1 and R2 were clearly visible (as shown in lane 4), indicating 0-0=00. When I1 was added to the solution, T2 was displaced, forming double-stranded waste, DNAzyme Z2 was activated, substrate R2 was cut, and the product O2 generated. However, DNAzyme Z1 was still combined with T1 and could not be activated, R1 still existed, and no product O1

generated. The gel band corresponding to R2 disappeared, and the gel band corresponding to O2 generated (as shown in lane 5), indicating 1-0=01. When input I2 was added to the solution, T1 and T2 were displaced. DNAzyme Z1 and Z2 were activated, substrates R1 and R2 were cut, and products O1 and O2 generated. Gel bands corresponding to R1 and R2 disappeared, and gel bands corresponding to O1 and O2 generated (as shown in lane 6), indicating 0-1=11. When input I1 and I2 were added to the solution at the same time, the two inputs inhibited each other, and Neither DNAzyme Z1 nor Z2 could be activated. Substrate R2 and R3 still existed, and no products O2 and O3 generated. The gel bands corresponding to R1 and R2 still existed (as shown in lane 7), indicating 1-1=00. The experimental results are in good agreement with the truth table, which verifies the accuracy of the experiment.

S2.3 4-bit open square root circuit

A 4-bit open square root circuit consists of an OR-INHIBIT gate, an AND-K gate and an OR gate. Four inputs S3, S2, S1, S0 indicate the binary number to be calculated, and outputs A1, A0 indicate the binary result of the square root. The output of OR-INHIBIT gate and AND-K gate indicates A0, and the result of OR gate indicates A1.

In order to verify the accuracy of the square root circuit, The result of gel electrophoresis experiment was shown in Figure S6(a). When calculating the floor of the square root of 0, no input was involved in the calculation system. Y3, Y5, Y6, R1 and R2 coexisted in the solution, and their corresponding gel bands were clearly visible (as shown in lane 3). In the calculation of the square root of 1, input S0 was added into the system, the OR-INHIBIT gate was activated and cut substrate R2, and O2 generated. The gel band corresponding to R2 disappeared, and the gel band corresponding to O2 formed (as shown in lane 4). When the square root result of 4 was calculated, input S2 was added to the system, the OR gate was activated and cleaved substrate R1, and product O1 was generated. The gel band corresponding to R1 disappeared, and the gel band corresponding to O1 formed (as shown in lane

5). When the square root result of 8 was calculated, input S3 was added to the system, the OR gate was activated and cleaved substrate R1, and product O1 generated. The gel band corresponding to R1 disappeared, and the gel band corresponding to O1 formed (as shown in lane 6). When the square root of 9 was calculated, input S3 and S0 were added to the system, the OR gate and the OR-INHIBIT gate were activated and cut substrates R1 and R2, O1 and O2 generated. Gel bands corresponding to R1 and R2 disappeared, and gel bands corresponding to O1 and O2 generated (as shown in lane 7). When the square root result of 12 was calculated, inputs S3 and S2 were added to the system. The OR gate and the AND gate were activated and cut substrates R1 and R2, and products O1 and O2 formed. Gel bands corresponding to R1 and R2 disappeared, and gel bands corresponding to O1 and O2 formed (as shown in lane 8).

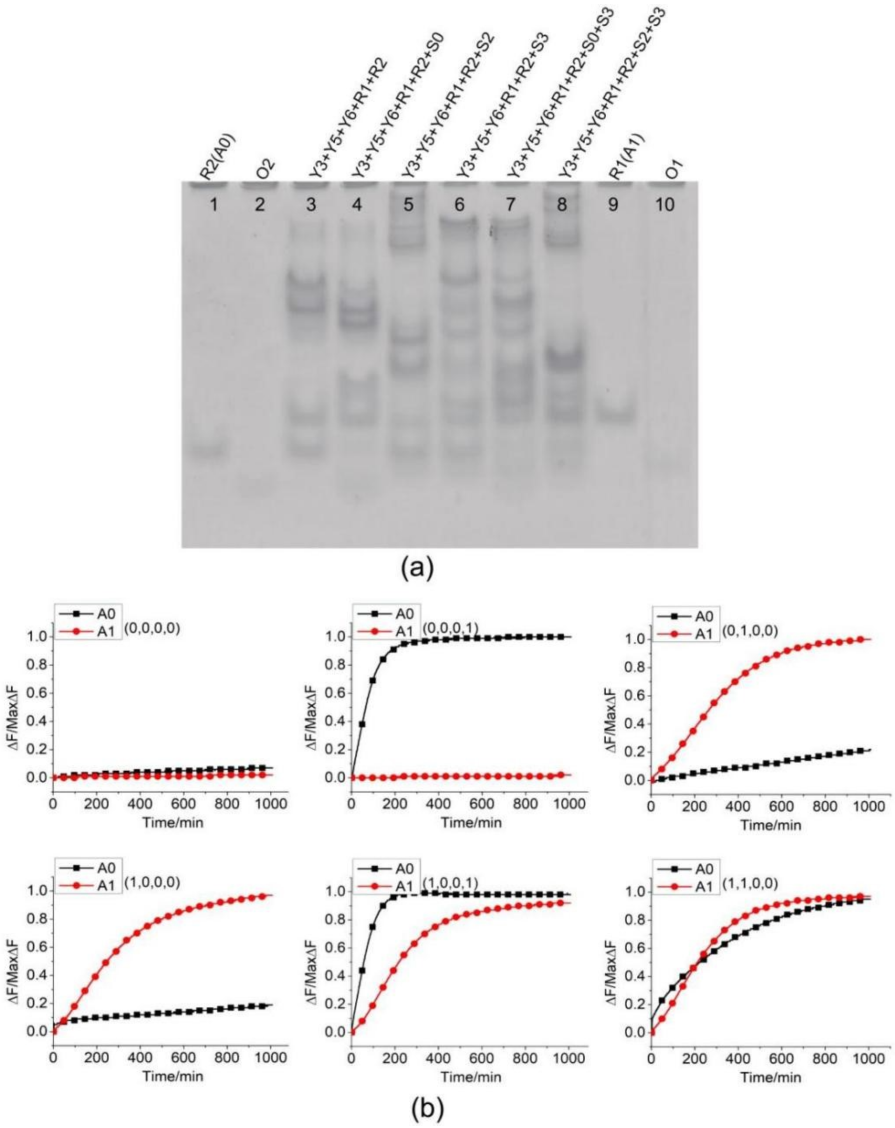

Figure S6. (a) Native PAGE analysis of the square root circuit products. The strands and complex involved were labeled above the lane number. Lane 3, gate complex Y3, Y5, Y6 and substrate R1, R2. Lane 4, products of the square root circuit triggered by input S0. Lane 5, products of the square root circuit triggered by input S2. Lane 6, products of the square root circuit triggered by input S3. Lane 7, products of the square root circuit triggered by input S0 and S3. Lane 8, products of the square root circuit triggered by input S2 and S3. [Y3]:[Y5]:[Y6]:[R1]:[R2]:[S0]:[S1]:[S2]:[S3]=1:1:1:1:1:1.2:1.2:3.6:2.4, [Y3]=0.5μM. (b) Time-dependent normalized fluorescence changes (ΔF/MaxΔF) during the calculation process. All data represent the average of three replicates. [Y3]:[Y5]:[Y6]:[R1]:[R2]:[S0]:[S1]:[S2]:[S3]=1:1:1:1:1:1.2:1.2:3.6:2.4, [Y3]=0.15μM.

The results of real-time fluorescence experiment were shown in Figure S6(b), which was consistent with the results of gel electrophoresis experiment. The square root circuit run correctly.

S3.DNA sequences

Table S1.DNA sequences

Index	Name	Sequences (5'->3')	Length（n.t.）
1	Z1	ACTGCTCAGCGATACGTTTCATGTGATGTAACGTCACCCATGTTAGTGAA	50
2	Z2	CACAGACAGCGATACGTTTCATGTGATGTAACGTCACCCATGTTCGTCAA	50
3	Z3	CACAGACAGCGATACGTTTCATGTGATGTAACGTCACCCATGTTCGTCAA	50
4	Z4	ACTGCTCAGCGATCCTTGGTTGGCTGTGATGATCTTCCCAAGGCACCCATGTTAGTGAA	59
5	Z5	CACAGACAGCGATCCTTGGTTGGCTGTGATCATCTTCCCAAGGCACCCATGTTCGTCAA	59
6	Z6	ACTGCTCAGCGATCCTTGGCTTGTATTGATGATCTTCCCAAGGCACCCATGTTAGTGAA	59
7	T1	GCCTTGGGAAGATGGTGTCATGGGTGACGTTACATCACATGA	42
8	T2	GCCTTGGGAAGATGGTGTCATGGGTGACGTTACATCACATGATGAGGAGCCAACCGAGGAACG	63
9	T3	GCCTTGGGAAGATGGTGTCATGGGTGACGTTACATCACATGATGAGGAGCCAACCGAGGAACG	63
10	T4	GGTGCCTTGGGAAGATGGTGTC	22
11	T5	ATGAGGAGCCAACCAAGGATCG	22
12	T6	ATGAGGAGCCAACGAAGGATCGC	23
13	T7	TCTGTCGGGTGCCTTGGGAAGAT	23
14	T8	CTGTCGGGTGCCTTGGGAAGATGGTGT	27
15	R1	TTCACTATrAGGAGCAGTTTTTTTTTTTTTTTTT	34

16	R2	TTGACGATrAGGTCTGTGTTTTTTTTTTTT	29
17	O1	GGAGCAGTTTTTTTTTTTTTTTTTT	25
18	O2	GGTCTGTGTTTTTTTTTTTT	20
19	I1	GACACCATCTTCCCAAGGCCTCATCATGTGATGTAACGTAACCCATG	47
20	I2	TCATGTGATGTAACGTCACCCATGACACTCCTTGGTTGGCTCCTCAT	47
21	I3	TCATGTGATGTAACGTCACCCATGACACTCCTT	33
22	I4	AAGGCCTCATCATGTGATGTAACGTAACCCATG	33
23	I5	GACACCATCTTCCCAAGGCTCCTTCGTTGGCTCCTCAT	38
24	S0	TCATGTGATGTAACGTCACCCATGACACTCCTT	33
25	S1	AAGGCCTCATCATGTGATGTAACGTAACCCATG	33
26	S2	GACACCATCTTCCCAAGGCTCCTTCGTTGGCTCCTCAT	38
27	S3	ATCTTCCCAAGGCACCCGACAGA	23
28	A0	GGTCTGTGTTTTTTTTTTTT	20
29	A1	GGAGCAGTTTTTTTTTTTTTTTTTT	25
